# A systematic study of work function and electronic properties of MXenes from first principles†

**DOI:** 10.1039/d2na00830k

**Published:** 2023-07-10

**Authors:** Khabib Yusupov, Jonas Björk, Johanna Rosen

**Affiliations:** a Division of Materials Design, Department of Physics, Chemistry, and Biology, Linköping University Linköping 581 83 Sweden khabib.yusupov@liu.se

## Abstract

Functional 2D materials are interesting for a wide range of applications. The rapid growth of the MXene family is due to its compositional diversity, which, in turn, allows significant tuning of the properties, and hence their applicability. The properties are to a large extent dictated by surface terminations. In the present work, we demonstrate the influence of termination species (O, NH, N, S, F, Cl, Br, I) on the changes in electronic structure, work function, dynamical stability, and atomic charges and distances of MXenes (Ti_2_C, Nb_2_C, V_2_C, Mo_2_C, Ti_3_C_2,_ and Nb_4_C_3_). Among these systems, the work function values were not previously reported for ∼60% of the systems, and most of the previously reported MXenes with semiconducting nature are here proven to be dynamically unstable. The results show that the work function generally decreases with a reduced electronegativity of the terminating species, which in turn is correlated to a reduced charge of both the metal and terminating species and an increased metal-termination distance. An exception to this trend is NH terminations, which display a significantly reduced work function due to an intrinsic dipole moment within the termination. Furthermore, the results suggest that halogen terminations improve the electrical conductivity of the materials.

## Abstract 2

Functional 2D materials are interesting for a wide range of applications.

## Graphical abstract

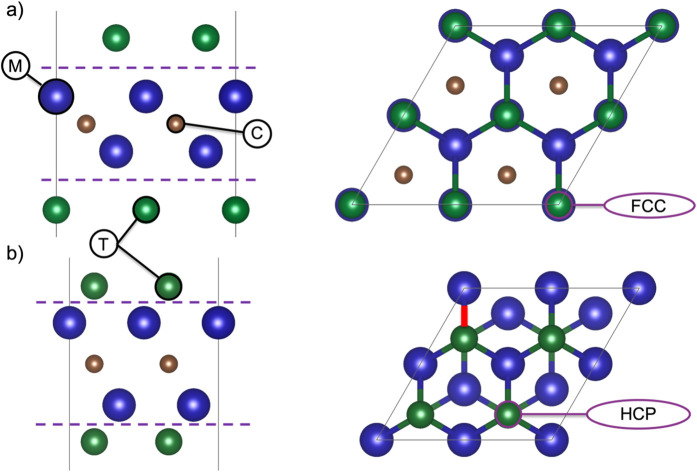

## Introduction

MXenes represent a group of two-dimensional (2D) inorganic compounds of the chemical formula M_*n*+1_X_*n*_T_*z*_ (*n*, *z* = 1–4), composed of *n* + 1 atomic layers of transition metal M (periodic table groups 3 to 6) with interlayers of carbon and/or nitrogen (X).^1^ T represents terminating species, commonly O, OH, and F, bonded to the outer layer transition metals. MXenes are typically synthesized by selective etching of A-layers, where A is an A-group element, commonly Al, from laminated MAX phase ceramics.^2,3^ The surface terminations can be tuned by choice of etching procedure and altered *via* removal of attained terminations with for example hydrogen exposure and heating.^4,5^ A change of the terminations affects the properties of the MXenes, which is of importance for tailoring the characteristics towards specific applications.^6,7^ To date, MXenes have shown high potential for electromagnetic shielding, energy storage, and more.^2,8^

MXenes are generally metallic, though theoretical predictions suggest that a change of termination can lead to a shift from a metallic to semiconducting nature.^9,10^ For example, Nb_2_CO_2_ is suggested to become a semiconductor by changing surface functionalization to nitrogen. Correspondingly, the MXene transparency can be tuned by choice of surface terminations, exemplified for Ti_3_C_2_T_2_,^11^ where a gain in transmittance of ∼77% was obtained when changing the MXene surface chemistry. Furthermore, the tunability of the work function with respect to termination makes MXenes potential candidates for field emitters or Schottky-barrier-free metal contacts to 2D semiconductors.^12^ Predictions for the hypothetical MXenes Cr_2_CO_2_ and Ti_2_C(OH)_2_ suggest values of the work function of ∼8 and ∼2 eV, respectively, which are the requirement for the most effective charge carrier injections (holes or electrons) into 2D semiconductors. Such MXenes could potentially be used as part of a metal–semiconductor junction.^13^

In a recent theoretical report^14^ the stability of six different MXenes (Ti_2_C, Nb_2_C, V_2_C, Mo_2_C, Ti_3_C_2,_ and Nb_4_C_3_) was studied as a function of surface terminations (O, OH, N, NH, NH_2_, SH, H, S, F, Cl, Br, I). It was shown that a majority of the terminated MXenes are stable in the form of single species terminations at full coverage for an appropriate chemical environment. The choice of atmosphere also dictates if an element prefers adsorbing in its elemental or hydrogenated form. The result of the work outlines the most significant (application-wise) and stable MXenes. Outstanding observations that remain to be experimentally verified include MXenes terminated by either NH or S, as well as the heavy halogens, such as Br and I, among others. Such terminations have been realized in layered structures of certain MXenes, but remain, to a large extent, to be realized in delaminated form. This comprehensive overview motivates a systematic study on the influence of the terminations on selected properties, for an increased fundamental understanding, and to serve as inspiration for verifying experimental investigations.

In the present work, the influence of surface terminations (O, NH, N, S, F, Cl, Br, I) on selected properties is investigated for six MXenes (Ti_2_C, Nb_2_C, V_2_C, Mo_2_C, Ti_3_C_2,_ and Nb_4_C_3_). The choice of terminating species is motivated by our previously identified most stable MXene-termination combination,^14^ and the herein presented systematic study includes both known^12,15^ as well as, property-wise, theoretically unexplored terminations. Some of the MXene/termination combinations were reported before, however, the information is scattered and hence was included in the current work to create a broad view. It should be stressed that the present work is not a review, and as such it does not discuss all previous reports in this and related areas. Instead, it is a systematic theoretical study that includes previously not reported data, while also allowing trends to be identified and discussed. The analysis of the work function, the density of states, band structure, elemental charges, and changes in the bond length is provided to exploit the impact of the choice of termination. For the MXenes studied herein, the work function investigation was performed mainly for Nb_2_C, Nb_4_C_3_, Mo_2_C, and V_2_C, leaving Ti_2_C and Ti_3_C_2_ MXenes almost untouched (both have previously been studied thoroughly). The influence on the work function is studied for NH, N, S, Cl, Br, and I terminations. In particular, the termination with NH results in extremely small values of the WF due to the presence of an internal dipole moment. A thorough examination of the dynamical stability was performed for all the MXenes studied in the present manuscript, to serve as inspiration for MXene synthesis and tuning of termination composition. It was established that most of the previously reported (hypothetical) MXenes with semiconducting properties are likely not dynamically stable and hence should not be prioritized in further studies.

## Computational methods

The calculation procedure for investigating MXene structures with various terminations was reported in.^14^ In short, all calculations were performed within the framework of density functional theory (DFT) using the Vienna *ab initio* simulation package (VASP).^16^ The projector-augmented wave (PAW) method^17^ was used together with a plane-wave energy cutoff of 400 eV, and the Perdew–Burke–Ernzerhow (PBE) functional was used to describe exchange–correlation effects. For the bare and the fully terminated MXenes, the respective in-plane lattice parameters were optimized for the 1 × 1 primitive unit cell, using a 24 × 24 *k*-point sampling. For calculation of the electronic structure, the systems were converged with respect to the *k*-point mesh, and the optimal mesh was found to be 48 × 48 × 1. Analysis of charge localization and charge transfer was performed using Bader analysis,^18^ and the work function (WF) energy was calculated as the difference between the electrostatic potential of the vacuum (vacuum potential) and the value of the Fermi level.

**Fig. 1:**
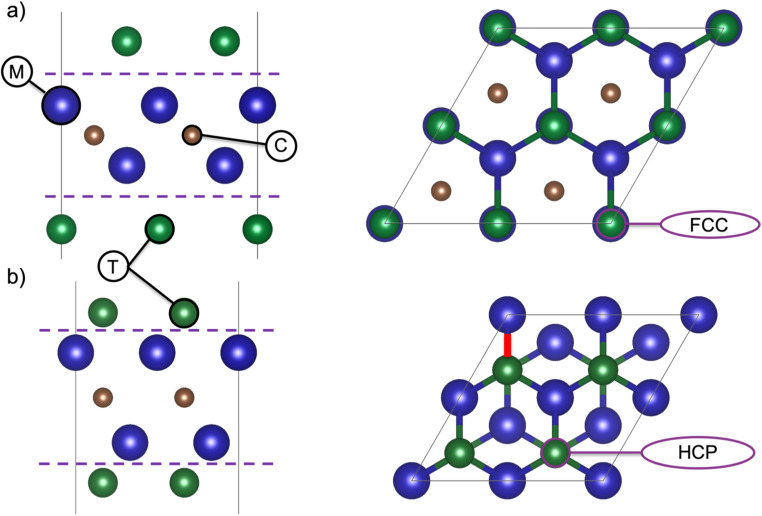
Schematic representation of M_2_XT_2_. The two different positions of termination species with respect to the M and X elements are shown for 2 × 2 supercells in a side view (left) and top view (right) for (a) FCC, and (b) HCP stacking sequence.

The unit cell represents a single MXene sheet, separated by a vacuum region of 17 Å perpendicular to the sheets. For all MXenes (*n* = 1–4), the position of the termination affects the properties, and Fig. 1 shows the two typical terminations sites for the M_2_CT_2_ structure. Fig. 1a and b represent the face-centered cubic (FCC) and hexagonal closed packed (HCP) hollow sites of the terminations, respectively. For the six MXenes investigated herein, each was studied for eight different terminations, the lowest energy positions of the terminating species are represented in Table S.1.†

## Results and discussion

### Influence of terminations on the work function, atomic charges, and interatomic distances

Based on the fact that the WF is affected by multiple characteristics of the material, we evaluate the change in the WF *via* monitoring the distances between termination atoms and the surface of MXene, charges of the atoms, *i.e.*, electron charge transfer, polarity, and electronegativity. Firstly, it should be noted that the WF is defined as the energy needed to remove an electron at the Fermi level of the material to infinity (the vacuum level). Thus, when the value of WF is being discussed it is given with respect to the Fermi level. The WF is highly sensitive to surface chemistry, especially for very thin materials. Furthermore, the WF is related to the dipole barrier, which, in turn, is correlated to electronegativity. In the case of surface adsorption, the WF depends on the electronegativity of the absorbates. These relations are well described in the work by Ramprasad *et al.*^19^ and Leung *et al.*^20^ Furthermore, for adsorbates with internal dipole moment (only NH in this study), the work function depends on the orientation of the adsorbate. Here, the NH-groups create interface dipoles pointing away from the surface of the MXenes, resulting in the work function being lowered. The electronegativity, size of the atoms, and overlap of the electron clouds (if the bonding is in place) affect the distance of the absorbates and charge redistribution, hence the WF changes can be monitored both by bond distances and charges.

**Fig. 2:**
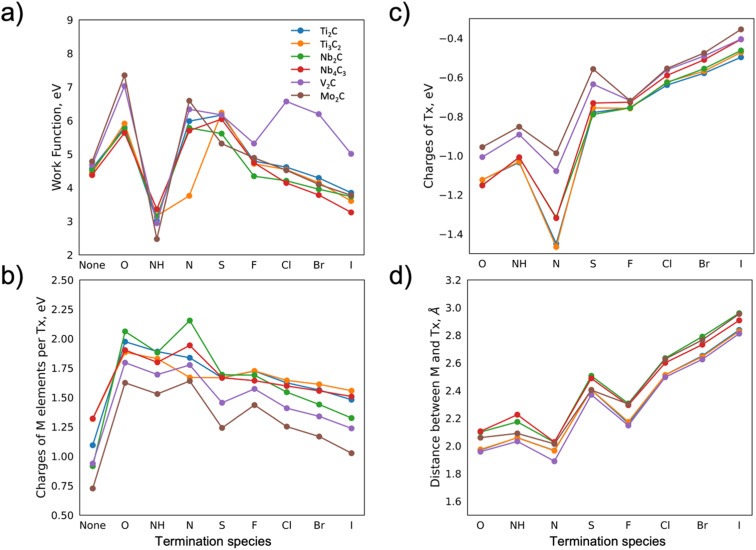
(a) Work function, (b) charge of the M elements, (c) charge of terminating species, and (d) distance between the M element and the closest termination as a function of termination species for various MXenes.

The WFs of the considered MXenes and the influence of the choice of termination are shown in Fig. 2a. As previously observed for Ti_3_C_2_ and V_2_C-based MXenes,^21^ the WF values are very sensitive to the terminating elements. Previously reported theoretical values of the WF, such as for Mo_2_CF_2_,^12^ and Ti_3_C_2_O_2_,^15^ were compared to those calculated herein, and no significant differences were found (within 3%). Minor deviations may be explained by the choice of pseudopotential used in the DFT calculations. There is a difference between experimentally and theoretically predicted values of the WF. For example, for Ti_3_C_2_O_2_, this difference is ∼20%,^15^ which may arise from surface contamination as well as mixed surface terminations that are challenging to take into account properly in the DFT calculations.

The trends in the change of work function for different terminations are similar for most MXenes. Going from oxygen to iodine the WF value is gradually decreasing, with an exception for NH terminations, for which the decrease is rather abrupt. This is likely linked to the change in electronegativity of the termination and the related distance between M and T (Fig. S.1†). For simplicity and motivated by the similar trends observed for the different MXenes, we choose to discuss the results for Mo_2_C, and then we extrapolate the argument to the other MXenes, where possible.

Comparing the hypothetical bare Mo_2_C with the oxygen-terminated one, the WF is drastically affected by the oxygen, from ∼4.7 eV (bare MXene) to ∼7.5 eV, respectively (Fig. 2a). This change in the WF can be explained by a charge transfer from the M elements towards X and, in particular, towards T. The charge transfer, in turn, creates a dipole moment (surface dipole or *D*_S_) between the terminations and the MXene surface. Charges for the X element for differently terminated Mo_2_C MXene are shown in Fig. S.2c,† showing no large variations compared to the changes observed for the M elements. Note that the net dipole moment of the system is zero. The *D*_S_ between the termination and the surface, however, varies for different terminations. If the electronegativity of termination is high, *i.e.*, it attracts more electrons, the formed negative charge of the *D*_S_ will point towards the surface of the MXene, leading to an increase in the WF. A nitrogen termination is one such example, where its presence leads to the WF value being as high as ∼6.7 eV.

The WF values decrease with a reduced electronegativity of the terminating elements along the *x*-axis in Fig. 2. The charge transfer from M to T, see Fig. 2b and c, is correspondingly reduced, hence the formed *D*_S_ is facing the surface of MXene with a smaller negative charge, leading to the decrease in WF value. For example, bromine and iodine terminations lead to WF values of ∼4.2 and 3.9 eV, respectively. The exception in the observed trend is the NH termination. The bonding between H and N leads to a lower total charge of termination as compared to pure N. Furthermore, the negatively charged N and positively charged H weaken the *D*_S_ and lead to a decrease in the WF (∼2.4 eV). The change in electronegativity for different terminations is also accompanied by a change in the distance between the M and T elements, which is also correlated to the WF. In general, the smaller the charge transfer, the larger distance, and the lower the WF. Comparing Fig. 2a and d, a correlation between a reduced WF and a larger distance between the M element and the termination is evident (apart from for NH which is discussed separately due to an internal dipole moment). It should be noted that the distance between the T and M elements is influenced by their atomic size, hence the atomic ratio T/M may also be correlated to the WF value.

The above-described trends are relevant for other MXenes, though with some variations. For example, for V_2_C MXene, we find higher values of the WF compared to the other MXenes for S, Cl, Br, and I terminations. The WF values for these terminations can be explained by various factors such as the high electronegativity of the terminating atoms alongside the atomic size of the vanadium atom, which is smaller compared to other M elements. For example, Cl, compared to Br and I terminations, exhibits the highest value of electronegativity. Alongside the quite high distance between the terminating atom and the surface of the bare MXene, and the unchanged value of the charge of the C atom, this leads to the increased dipole moment *D*_S_, and hence the higher value of the WF. Further, one can see that both the decrease in electronegativity for Br and I atoms and the increase in the distance between the terminations and the surface result in lower values of the WF.

Another exception is Ti_3_C_2_ terminated with N. The WF value for this system is rather low (∼3.7 eV) and close to the result for NH terminations (∼3.2 eV). Notably, the low value is accompanied by a small distance between the N and C (Fig. S.2b†), though no further investigation is performed on this system as it is found to be dynamically unstable (see below).

The WF, although being an important MXene property, is not often investigated. To the best of our knowledge, we here (Table S.2†) summarize the values for previously reported as well as unexplored structures (48 structures in total, 28 not yet reported). The table includes the WF of previously reported systems for which the WF was studied. The table therefore also indicates the currently missing theoretical data for specific MXenes. The value of the work function is crucial for the field of semiconductors, exemplified by the introduction of MXene into perovskite, which led to a Schottky barrier due to the difference in WF and, as a result, a boosted solar energy conversion.^22^

### The effect of terminations on electronic properties

Surface terminations impact the electronic structure. Though semiconducting MXenes could be of great interest for electronic applications, the reports of semiconducting MXenes are scarce, motivating our choice to partially center the discussion around the properties of such systems. It should be noted that though some of the MXenes with associated terminations were reported before, the full impact of terminations on the electronic structure is scattered, and hence the analysis of also previously reported systems will be included in the present manuscript. The list of all structures is presented in Table 1. It should be noted that many theoretical studies on MXenes with and without terminations do not include an evaluation of stability, *i.e.*, if the material is likely to be synthesized or not. We have therefore performed an evaluation of dynamical stability (Fig. S.3†) for all systems included in the present study and show that some of the previously reported and here identified semi-metallic systems are not likely to be experimentally obtained, indicated by the presence of frequencies below zero in the spectra. This information is crucial for future research from both a theoretical and experimental perspective. Therefore, all structures predicted to be unstable have been excluded from a more detailed discussion and further evaluation. For the MXenes calculated to be dynamically unstable it should be noted that this does not necessarily mean that those MXenes cannot be synthesized, but that they might be stabilized by affects not taken into consideration by our calculations. *E.g.*, it has been shown that the ideal Mo_2_CF_2_ MXene can be stabilized either by charge density waves resulting in a Peierls-like distortion of the ideal structure, or, alternatively by magnetic effects, requiring theory beyond the generalized gradient approximation. Such effects concomitantly alter the electronic band structure.^23^ Notably, many “ideal” systems investigated in the literature have not been experimentally verified, *i.e.*, going into a detailed discussion on individual materials and appropriate methods for predicting their stability is beyond the scope of our study, since we are interested in general trends for how surface terminations affect MXenes. Therefore, we limit our study to MXenes represented in their ideal primitive unit cell, and leave the detailed examination of MXenes calculated to be dynamically unstable for future studies.

**Table 1:** The effect of choice of termination on the appearance of energy/band gaps in the electronic structures of 6 different MXenes, given by the notation metallic (M.) and semi-metallic (S.M.)^a^

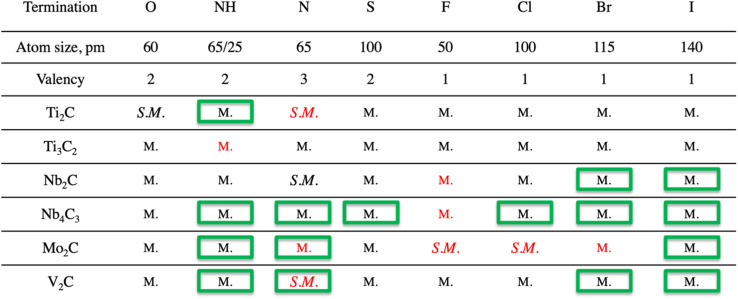

a The structures marked by a green box are those previously not reported in the literature. The structures that are marked with red color are found to be dynamically unstable.

Most of the systems investigated in this study exhibit metallic-like electronic structures, *i.e.*, there are no energy gaps close to the Fermi level, and hence electrons can move freely through the structure. It should be noted that close to bare MXene has to date only been realized under vacuum,^4^ and is highly challenging to be attainable in air. Still, the electronic structure of pure MXenes is a powerful tool to establish the influence of terminations over the MXene properties.^9^ For example, there is an energy gap in the region of −2 eV in non-terminated Ti_2_C MXene. Further adsorption of elements or terminations leads to changes in the electronic structure and depending on the choice of T, the resulting MXene (terminated) can exhibit a metallic or semiconductor nature, as previously reported.^24–27^ The reason behind the transition from a metallic to semiconductor nature lies in the position of the Fermi level and hybridization between p orbitals of the termination and d orbitals of M.^9^ For the pure MXenes (those that exhibit metallic nature) the Fermi level is close to the d bands of the M element. However, further hybridization creates new bands below the Fermi level and shifts the latter to the center of the energy gap that originates from the separation of the bands from the X and M elements.

It should be noted that the semiconducting nature, once achieved, has to be carefully evaluated and characterized. For example, the energy gap can vary with the implementation of additional parameters like spin–orbital coupling. Another example is that for certain applications, it is important whether the band gap is direct, *i.e.*, the excited electron can emit a photon, or indirect, *i.e.*, additional phonon assistance is required.

Among the previously reported MXene systems and those presented in this study, there are a few that exhibit a semiconductor nature, *i.e.*, the presence of bandgap. The values of all energy gaps are listed in Table S.3.† Ti_2_CT_*z*_, for example, exhibits band gaps of ∼0.33 and ∼1.0 eV for O and N terminations, respectively. Note that the values of these gaps are obtained by PBE generalized gradient exchange–correlation functional, known to underestimate the width of the gaps, and as such should be used to study trends rather than the specific values. The electron configuration of oxygen allows two electrons to be accepted in the outermost orbitals (p), which shift the Fermi level significantly so that the energy gap is recognized as a bandgap, implying that more electrons may be transferred from the pure MXene to the termination to attain an increased gap.^28^ For example, the same MXene with F terminations does not exhibit a bandgap, which can be explained by the p orbital (outermost orbital) of fluorine accepting only one electron.

**Fig. 3:**
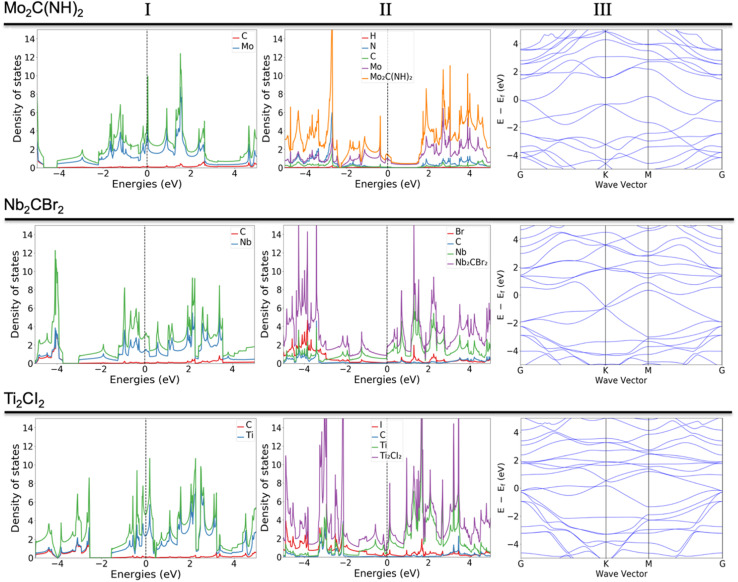
Column (I) DOS of pure MXenes, column (II) DOS of MXenes with adsorbed elements; column (III) band structures associated with column (II). Electronic structure (DOS and band structures) for Mo_2_C(NH)_2_ in the first row, Nb_2_CBr_2_ in the middle row, and Ti_2_CI_2_ in the bottom row.

Some of the previously not reported MXenes with specific terminations are of high potential interest for application in various fields. Those that deserve more attention or represent a common behavior upon termination adsorption are presented in Fig. 3. The termination of NH groups is quite uncommon for MXenes. While atomic nitrogen tends to create band gaps within MXenes, which could find vast use in applications within the semiconductor field, the influence of NH in the current study shows extraordinary behavior for some systems (Fig. 3, row a). For example, the termination of Mo_2_C MXene with NH results in the d orbitals (d_*xz*_, d_*xy*_, d_*yz*_) of Mo atoms being dominant around the Fermi level, which is clearly observable from both total and partial DOS (Fig. S.1A†); the carbon and NH groups are mostly contributing to the band structure through p orbitals either significantly above the Fermi level or starting from −1.5 eV and downwards. Very similar behavior was found in previous work^29^ for the hypothetical O-terminated W_2_C MXene. There it was found that the band structure did exhibit semi-metallic nature (semiconductor with zero bandgaps), however, the addition of extra parameters such as spin-orbital coupling (SOC) and/or utilization of hybrid functional led to the formation of a small band gap. The very same pattern of the electronic structure and similar contribution of various d and p orbitals indicates that NH-terminated Mo_2_C MXene may exhibit a semiconductor nature with the application of more precise calculations, *i.e.*, the shift of the bands close to the Fermi level and appearance of an energy gap. Indeed, a closer comparison of the two systems shows that both terminations exhibit two available electron orbitals in the outermost shell, though for NH it is a result of the bonding with hydrogen.

The terminations with Br and I (Fig. 3, rows b and c) were expected to give different behavior due to the larger atomic radius and the presence of d orbitals, which should affect the hybridization and contribution to the overall DOS. Indeed, both terminations contribute quite differently compared to the more conventional terminations. Due to the presence of d orbitals of T, the p orbitals are located near the Fermi level (both above and below). There are well-distributed bands from all elements of the MXene, including terminations, near the Fermi level, which indicates that the system exhibits a metallic nature. One can assume that terminations exhibiting d-orbitals are a powerful tool to boost the transfer of charges, *i.e.*, resulting in widening the range of applications.

Changes in the form of a transition from metal to semiconductor were here demonstrated for MXenes exhibiting the M_2_X composition, *i.e.*, the strong influence from a choice of termination is at least in part due to the ratio between M and T (1 : 1). Once the ratio is shifted to 3 : 2 or 4 : 2 the influence is less significant. For example, none of the Ti_3_C_2_ or Nb_4_C_3_ MXenes showed any major change in the electronic structure for different terminations (no transition from metallic to semimetallic or semiconductor).

Some general observations can be made. MXene terminations are surely a powerful tool to change the MXene properties, however, the thicker the system (*i.e.* more layers of M and X), the smaller the impact: The termination of M_2_X MXenes leads to major changes in properties such as potential transitions from metals to semiconductors and presence of additional energy gaps. The terminations exhibiting higher valency tend to create stronger hybridization with the M elements and form band gaps. The more electrons needed to fill the orbitals of T, the larger the gap tends to be. The terminations that possess high energy states reinforce the electronic structure by adding more electronic states.

### Applicability of selected MXenes

The current work does not discuss the latest synthesis routes for MXenes nor the details of different synthesis techniques. There are structures that are mentioned in the work that were previously theoretically predicted or/and experimentally verified, however, evaluation thereof is not within the scope of the work. The discussion is carried out within the frame of the applicability of MXenes in various fields.

Described changes of charge, WF, and electronic structure with coupled terminations are valuable information for various fields of applications. The difference between the exhibited properties of the same MXene with altered terminations is so diverse that one system can be applied in multiple fields. For example, in energy-related fields such as solar and thermal energy conversion, MXenes that either exhibit semiconducting nature or metallic nature with low WF can be vastly applied.

Thermal conversion, *i.e.*, the direct conversion of heat into electricity, is of high interest since the driving force of this phenomenon (energy difference) is passim.^30,31^ In this application, low thermal conductivity and high electrical conductivity are of importance, hence MXenes are potential candidates for the field of thermoelectrics (TE).^32^ The Ti_2_CO_2_ exhibits the figure-of-merit *ZT* = 0.45 and 0.27 for n-doping and p-doping, respectively. For the maximum *ZT* to be reached, one of the requirements is the presence of a quite narrow bandgap.

The high electrical conductivity of MXenes is a desirable property for other applications such as biosensors^33^ and hydrogen and oxygen evolution reaction (HER/OER).^34–36^ If the MXene is conductive under the standard condition, it can transfer charges during the evolution reaction, which is one of the main requirements. With this in mind, the Br and I termination could be of high value due to their effect on the electronic structure of the MXene.^37^

For the actual specific application, more thorough and precise calculations are required. For example, the utilization of resource-demanding computations, such as the use of hybrid functionals, is required to be able to compare the calculated bandgap with corresponding experimental values. Nevertheless, the current study provides concentrated/gathered information regarding the influence of already known and completely new configurations of MXene with various terminations. Further research should be focused on in-depth theoretical analysis of here identified stable novel systems, and related experimental exploration is encouraged.

## Conclusion

We have investigated the influence of eight termination species on the properties of six MXenes with density functional theory computations. For each combination of MXene and termination, the dynamical stability was investigated, showing that among the total of 48 structures, there are 39 that are dynamically stable, and 14 which are newly reported ones. Furthermore, the work function, electron structures (DOS and band structures), distances between atoms, and influence of termination species on the atomic charges were thoroughly investigated.

Considering work function, it is closely related to the valency of the termination species, atomic sizes of the atoms, electronegativity, and the distance between the termination atoms and the surface of the MXene. The valency directly affects the charges of the termination species and results in the formation of a dipole moment between terminations and the surface of the MXene. This surface dipole creates an energy barrier for the electrons to be withdrawn from the surface of the MXene. The bigger the distance between the termination species and the MXene surface, the weaker effect of the dipole moment, resulting in smaller WF. The internal dipole moment of the NH group results in a surface dipole directed in the opposite orientation compared to the other terminations in this study. The NH termination hence lowers the value of WF, with the lowest value being ∼2.4 eV for the Mo_2_C(NH)_2_ system.

The analysis of the electronic structures of MXene with respect to termination species showed that most of the studied structures exhibit a metallic nature. For those structures that exhibit a semiconductor nature, the mechanism behind the shift is mainly due to valency and hybridization that creates the energy gap. The terminations with high energy states (d orbitals) affect the hybridization differently, resulting in the well-distributed contribution of termination states into the overall DOS of the MXene. Such elements (Br, I) tend to reinforce the region near the Fermi level with extra bands, hence increasing the electrical conductivity.

Possible applications of new MXene chemistries are suggested. Metallic MXenes with very high or low values of WF can be used in tandem with 2D semiconductors to effectively inject charge carriers (holes or electrons). The MXenes electronic structures that are reinforced by d orbitals of termination species can be of high importance for sensors and in fields where high electrical conductivity is needed. MXenes with a semiconductor nature could be of interest for various energy applications, such as solar cells, transistors, thermoelectric materials, and others. Further research on these materials from both a theoretical and an experimental perspective is encouraged.

## Conflicts of interest

There are no conflicts to declare.

## Supplementary Material

NA-005-D2NA00830K-s001
