# Supplementary material for: A systematic study of work function and electronic properties of MXenes from first principles
Source: Nanoscale Adv. 2023 Jul 10;5(15):3976–84. doi: 10.1039/d2na00830k (PMC10367962; doi:10.1039/d2na00830k)
Supplement: NA-005-D2NA00830K-s001 [file NA-005-D2NA00830K-s001.pdf]

Electronic Supplementary Material (ESI) for Nanoscale Advances.
This journal is © The Royal Society of Chemistry 2023

Support information

A systematic study of work function and electronic properties of MXenes from first principles

Khabib Yusupov[1], Jonas Björk[1], Johanna Rosen[1]

[1]Division of Materials Design, Department of Physics, Chemistry, and Biology, Linköping University, Linköping, Sweden, 581 83

Table S. 1. Position of the terminating species with respect to the M and X elements of the MXene.

	Type of cite (FCC or HCP) with respect to T_z							
Structures	O	NH	N	S	F	Cl	Br	I
Ti2C	FCC	FCC	HCP	FCC	FCC	FCC	FCC	FCC
Ti3C2	FCC	FCC	HCP	FCC	FCC	FCC	FCC	FCC
Nb2C	FCC	HCP	FCC	FCC	FCC	FCC	FCC	FCC
Nb4C3	FCC	HCP	FCC	HCP	HCP	HCP	HCP	HCP
Mo2C	HCP	HCP	HCP	HCP	FCC	FCC	FCC	FCC
V2C	FCC	FCC	FCC	FCC	FCC	FCC	FCC	FCC

Table S. 2. MXenes with corresponding T_Z. The represented values of WF are only those that were reported before in the literature[12,37]. The green frame reflects unreported MXene/T_Z combinations and their WF. Empty slots are of the reported structures but no WF data.

Termination	O	NH	N	S	F	Cl	Br	I
Presence of work function values								
Ti_2C	5.80	3.02		6.03	5.04	4.64	4.32	3.81
Ti_3C_2	6.16			6.15	4.98	4.50	4.11	3.56
Nb_2C	5.81				4.48		3.95	3.73
Nb_4C_3	5.77	3.36	5.69	6.04	4.97	4.14	3.78	3.26
Mo_2C	7.39	2.47	6.58		5.74			3.77
V_2C	6.68	2.94	6.33		5.52		6.19	5.01

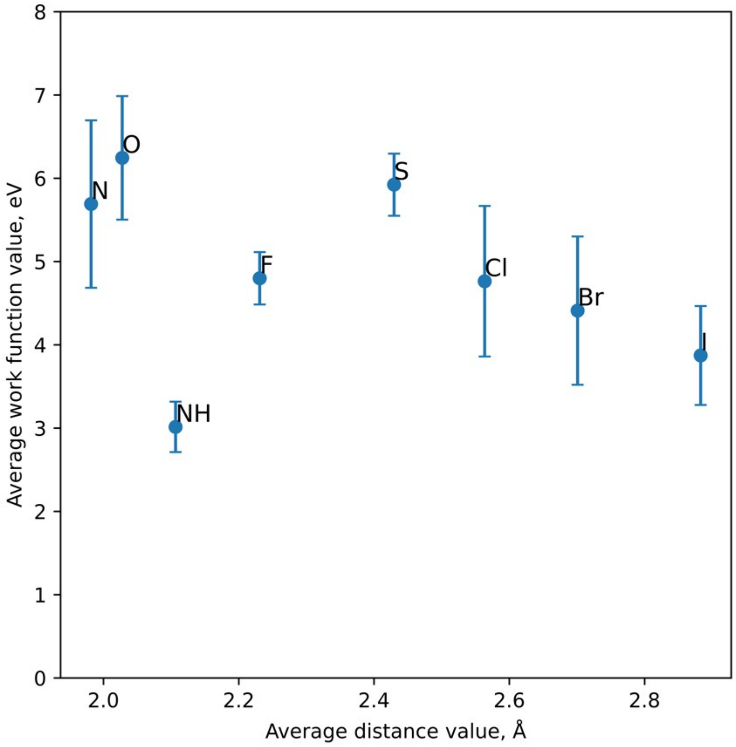

Figure S. 1. Relation between the average work function and the average distance between the M element and the closest termination. The values are averaged over the data for the 6 studied MXenes presented in Figure 2a and 2d of the main paper. The graph confirms the general correlation between a reduced WF and a larger distance between the M element and the termination (apart from for NH which is discussed separately due to an internal dipole moment).

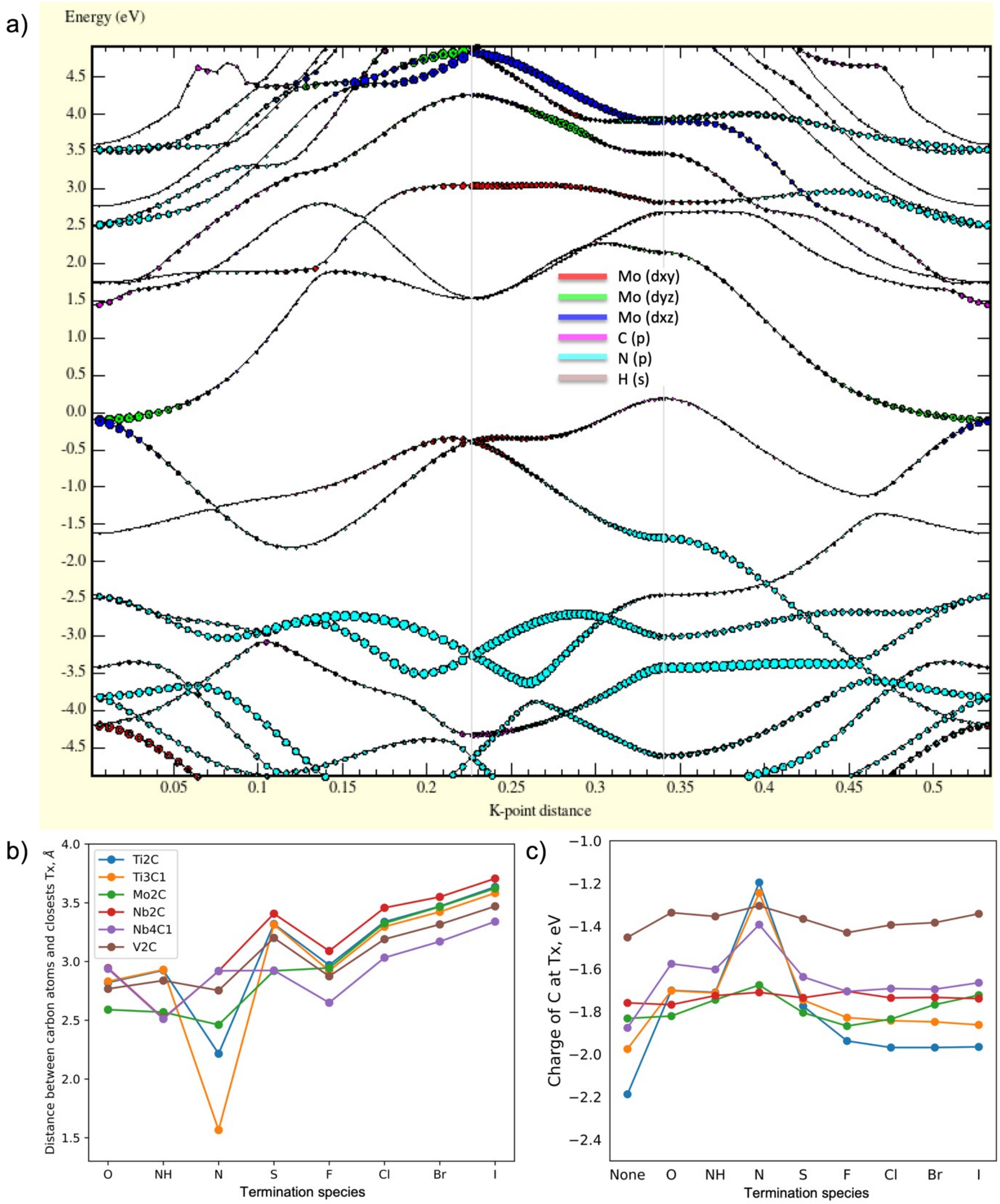

Figure S. 2. a) Band structure of $Mo_2C(NH)_2$ MXene with projected bands of Mo d- and T_z p/s-orbitals; b) influence of T_z elements on the distance between X and T_z distance c) Charges of carbon atoms for the MXenes with associated terminations

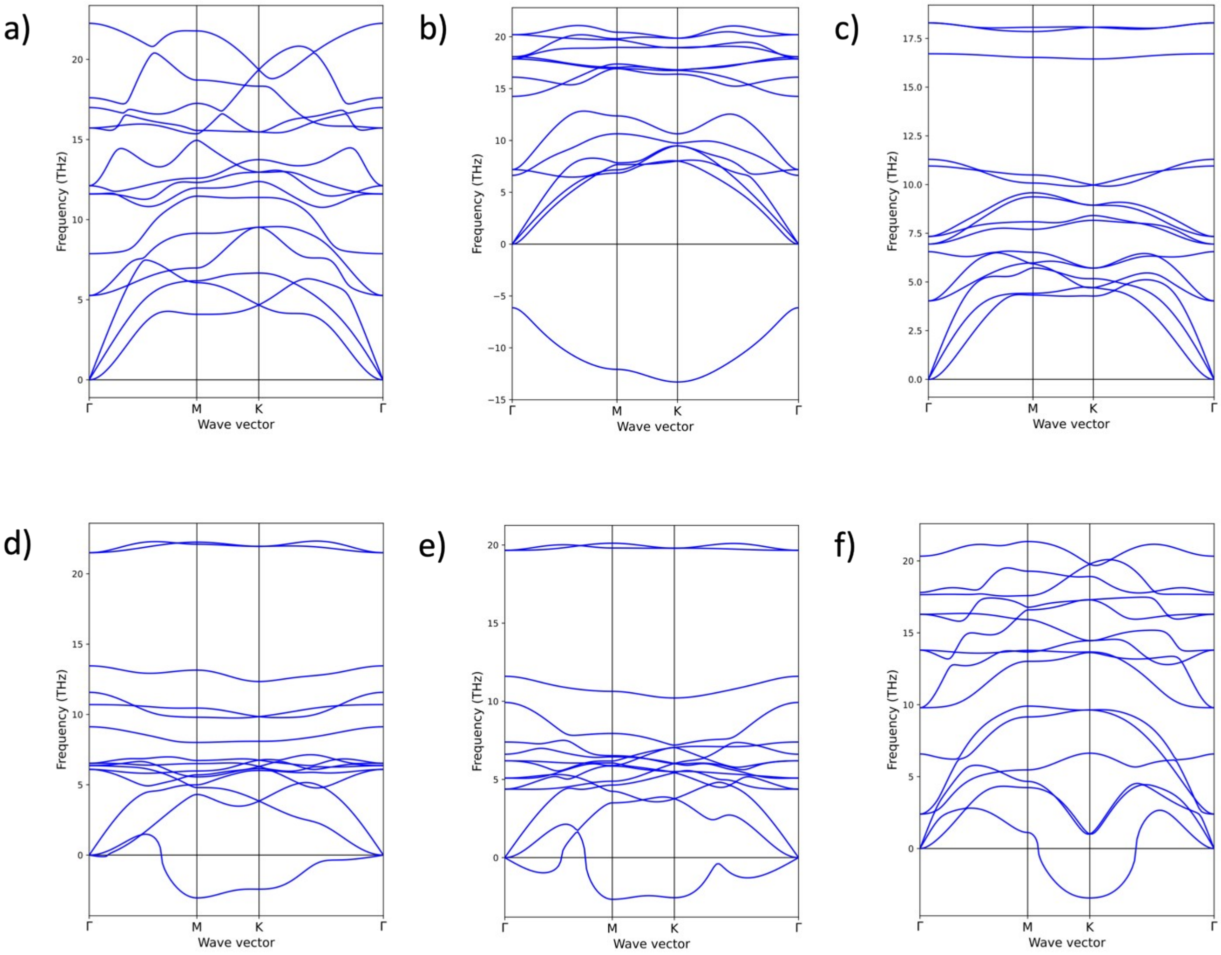

Figure S. 3. Phonon dispersion for a) Ti_2CO_2, b) Ti_2CN_2, c) Nb_2CN_2, d) Mo_2CF_2, e) Mo_2CCl_2, and f) V_2CN_2

We have included the phonon spectra of the MXenes with predicted semiconducting nature. These results highlight that most semiconducting MXenes are not obtainable, and further analysis is therefore not being pursued, and attempts for experimental verification is not recommended. The phonon spectra of Ti2CO2 and Nb2CN2 exhibit no negative frequencies and are therefore considered to be dynamically stable.

Table S. 3. Energy gaps of Semi-metallic MXenes. The structure marked by green is the one that was not reported in the literature previously.

MXene	Ti_2CO_2	Ti_2CN_2	Nb_2CN_2	Mo_2CF_2	Mo_2CCl_2	Nb_2CN_2
Energy gap, eV	0.33	1.0	0.2	0.24	0.11	0.211
